# Cardiac Specific Expression of Threonine 5 to Alanine Mutant Sarcolipin Results in Structural Remodeling and Diastolic Dysfunction

**DOI:** 10.1371/journal.pone.0115822

**Published:** 2015-02-11

**Authors:** Mayilvahanan Shanmugam, Dan Li, Shumin Gao, Nadezhda Fefelova, Vikas Shah, Antanina Voit, Ronald Pachon, Ghassan Yehia, Lai-Hua Xie, Gopal J. Babu

**Affiliations:** Department of Cell Biology and Molecular Medicine, New Jersey Medical School, Rutgers, The State University of New Jersey, Newark, New Jersey, United States of America; Universidade Federal do Rio de Janeiro, BRAZIL

## Abstract

The functional importance of threonine 5 (T5) in modulating the activity of sarcolipin (SLN), a key regulator of sarco/endoplasmic reticulum (SR) Ca^2+^ ATPase (SERCA) pump was studied using a transgenic mouse model with cardiac specific expression of threonine 5 to alanine mutant SLN (SLNT5A). In these transgenic mice, the SLNT5A protein replaces the endogenous SLN in atria, while maintaining the total SLN content. The cardiac specific expression of SLNT5A results in severe cardiac structural remodeling accompanied by bi-atrial enlargement. Biochemical analyses reveal a selective downregulation of SR Ca^2+^ handling proteins and a reduced SR Ca^2+^ uptake both in atria and in the ventricles. Optical mapping analysis shows slower action potential propagation in the transgenic mice atria. Doppler echocardiography and hemodynamic measurements demonstrate a reduced atrial contractility and an impaired diastolic function. Together, these findings suggest that threonine 5 plays an important role in modulating SLN function in the heart. Furthermore, our studies suggest that alteration in SLN function can cause abnormal Ca^2+^ handling and subsequent cardiac remodeling and dysfunction.

## Introduction

Sarcolipin (SLN), a 31 amino acid sarco/endoplasmic reticulum (SR) membrane protein is expressed predominantly in atria and in skeletal muscles and to a very low level in the ventricles [1]. The role of SLN as an inhibitor of cardiac SR Ca^2+^ ATPase (SERCA) is established by overexpressing SLN in the adult rat ventricular myocytes [2] and in mouse hearts by transgenesis [3–5]. Results from these studies have demonstrated that increased levels of SLN can inhibit the SERCA function and impair the myocyte contractility. The functional relevance of SLN expression in atria was elucidated by using a gene knockout mouse model [6]. Ablation of SLN resulted in an increase in atrial SERCA function and contractility [6]. However, the constitute activation of atrial SERCA pump due to SLN ablation resulted in electrophysiological and structural remodeling [7]. Together these studies indicate that SLN plays a key role in maintaining the atrial SERCA function and subsequently Ca^2+^ homeostasis and muscle contractility.

Altered levels of SLN mRNA and protein have been reported in humans and in animal models of heart diseases. The expression levels of SLN mRNA [8] and protein [9] were shown to be downregulated in atria of patients with atrial fibrillation. Sarcolipin protein expression was increased in the atrial myocardium of a dog model of pacing induced heart failure, whereas SLN protein level was decreased in atria of ischemic myocardium [1]. We have recently shown that SLN protein level was significantly increased in the ventricles of patients with mitral regurgitation [10] and in animal models of volume overload cardiac hypertrophy [11]. These studies along with studies using transgenic (TG) mouse models [3–5] suggest that in the diseased myocardium, changes in SLN level can affect SERCA function and calcium homeostasis. However, mechanisms other than the changes in the expression levels which modulate SLN function in the heart have not been fully understood.

It has been shown that both transmembrane and luminal domains of SLN are involved in the interaction and inhibition of SERCA pump [12–14]. Studies have also shown that SLN and phospholamban (PLN) can form heterodimers, which have a superinhibitory effect on the SERCA pump [15]. On the other hand, cardiac specific expression of SLN in the PLN knockout mice have demonstrated that SLN can function independently of PLN and can mediate the β-adrenergic receptor signaling in the heart [5]. Consistent with these findings, SLN null atria show a blunted response to isoproterenol (ISO) stimulation [6]. Together, these studies suggest that the β-adrenergic receptor signaling can modulate SLN function in the heart.

Using heterologous co-expression systems [16] and adult rat ventricular myocytes [17], it has been demonstrated that the conversion of threonine 5 (T5) to glutamic acid (T5E) at the N-terminus of SLN resulted in the loss of its inhibitory effect; whereas, T5 to alanine (T5A) mutation enhances its inhibitory effect. Furthermore, it has been demonstrated that T5 can be phosphorylated by serine threonine kinase 16 [14] or by calcium-calmodulin dependent protein kinase II (CaMKII) *in vitro* [17]. A recent structural study suggests that T5 can interact with SERCA at Trp392, and phosphorylation of the T5 can destabilize the binding of SLN to SERCA pump [18]. Together these studies suggest that T5, which is conserved among mammals [19], could play an important role in modulating SLN function. To address the *in vivo* role of T5 in modulating SLN function, a TG mouse model with cardiac specific expression of threonine → alanine (T5A) mutant SLN was created to abrogate SLN phosphorylation and its role in cardiac muscle contractility was studied. Results presented in this study demonstrate that the cardiac specific expression of SLNT5A results in severe atrial pathology and diastolic dysfunction.

## Materials and Methods

### Ethics Statement

All experiments were performed in accordance with the provision of the animal welfare act, the PHS policy on Human Care and Use of Laboratory Animals, and of AAALAC International and the guidelines and policies approved by the Institute Animal Care and Use Committee (IACUC) in the New Jersey Medical School (NJMS), Rutgers, Newark, NJ. For tissue harvesting, animals were euthanized by injecting pentobarbital following approved IACUC protocol.

### Generation of transgenic mice

The N-terminally FLAG-tagged mouse T5A mutant SLN (NF-SLNT5A) cDNA was generated by polymerase chain reaction (PCR) and cloned into the mouse α-myosin heavy chain (αMHC) transgenic promoter vector. To generate the transgenic founder mice, the transgene construct was microinjected into the male pronuclei of FVBN murine embryos at the transgenic core facility at NJMS, Newark. Mice carrying the transgene were identified by PCR analysis using primers specific for αMHC and SLN cDNA as described earlier [3].

Histopathological analysis

Five-μm paraffin sections of atrial and ventricular tissues from one- month and six-month old TG and non-transgenic (NTG) mice were stained with Hematoxylin and Eosin (H&E) and Masson’s trichrome following standard procedures. Quantitation of fibrotic area was calculated using NIH ImageJ 1.43u program.

Western blot analysis

Total protein extracts from the atrial and ventricular tissues were used for standard Western blot analyses. Briefly, equal amounts of total protein extracts separated on the sodium dodecyl sulfate-polyacrylamide gels (SDS-PAGE) were transferred to nitrocellulose membranes and probed with antibodies specific for SLN (anti-rabbit, 1:3000), SERCA2a (anti-rabbit, 1:10,000), triadin (anti-rabbit 1:5000), PLN (anti-rabbit 1:5000)[1], calsequestrin (CSQ; anti-rabbit, 1:5000, Affinity BioReagents), ryanodine receptor 2 (RyR2; anti- mouse, 1:500, Thermo Scientific), dihydropyridine receptor α(DHPRα; anti- rabbit, 1:1000, Thermo Scientific), sodium-calcium exchanger (NCX; anti-mouse, 1:500, Swant), 20Sα5 (anti-rabbit, 1:100), 20Sβ2 (anti-mouse, 1:1000), Rpt1(anti-mouse, 1:5000), Rpn2 (anti-mouse, 1:5000), 11Sα (anti-rabbit, 1:1000), 11Sβ (anti-rabbit, 1:1000) and glyceraldehyde 3-phosphate dehydrogenase (GAPDH; G8795, anti-mouse, 1:10,000, Sigma). Signals detected by Super Signal WestDura substrate (Pierce) were quantitated by densitometry and then normalized to GAPDH levels.

SR Ca^2+^ uptake assays

SR Ca^2+^ uptake was measured in the atrial and ventricular homogenates by the Millipore filtration technique as described earlier [3]. Briefly, the tissues were homogenized in 8 volumes of protein extraction buffer (in mmol/L, 50 KP_i_, 10 NaF, 1 EDTA, 300 sucrose, 0.5 dithiothreitol, and 0.3 phenylmethylsulfonyl fluoride). About 150 μg of the total protein extract was incubated at 37°C in 1.5 ml of Ca^2+^ uptake medium (in mmol/liter, 40 imidazole, pH 7.0, 100 KCl, 5 MgCl_2,_ 5NaN_3_, 5 potassium oxalate, and 0.5 EGTA) and various concentrations of CaCl_2_ to yield 0.03–3 μmol/liter free Ca^2+^ (containing 1 μCi/μmol ^45^Ca^2+^). To obtain the maximal stimulation of SR Ca^2+^ uptake, 1 μm ruthenium red was added immediately prior to the addition of the substrates to begin the Ca^2+^ uptake. The reaction was initiated by the addition of 5 mmol ATP and terminated at 1 min by filtration. The rate of SR Ca^2+^ uptake and the Ca^2+^ concentration required for half maximal velocity of Ca^2+^ uptake (EC_50_) were determined by non-linear curve fitting analysis using Graph Pad PRISM 4.0 software.

### Echocardiography and hemodynamics

In brief, mice were anesthetized with 2.5% tribromoethanol and echocardiography was performed using the high resolution ultrasound machine VisualSonic/Vevo 770 system with a high frequency transducer (30MHz) as described [20]. Left ventricular (LV) dimensions, wall thicknesses, LV fractional shortening (FS), and LV ejection fraction (EF) were measured from LV M-Mode images. Left atrium anterior-posterior dimension was measured from LV long-axis view. LV inflow through mitral valve was recorded by pulse-waved Doppler. Maximal velocity of E and A waves were measured for LV diastolic function and left atrial function evaluation. For β-adrenergic receptor stimulation studies, ISO at 0.02 μg/Kg/min was infused into the myocardium of 3–4 month old NTG and TG mice via jugular vein using an infusion pump at 2μl/min for five minutes followed by the dose of 0.04μg/Kg/min. 2D and M-mode echocardiographic images were obtained at baseline and after five minutes of each dose. For hemodynamic studies, the pressures in the LV and abdominal aorta were measured simultaneously using two separate 1.4F Millar catheters and the pressure gradients were calculated.

### Proteasome Assay

Chymotryptic activity of the proteasome was measured in atria and in the ventricles of one-month old mice as described [21]. Briefly, 30 μg of total protein extract in 1 ml assay buffer containing (in mmol/L) 25 HEPES, pH 7.5, 0.5 EDTA, and 40 fluorogenic substrate, Suc-LLVY-AM (Boston Biochem, Cambridge, MA) was incubated at 37°C for 2 hrs in the presence of ATP and the fluorescence was measured (Turner Designs, Sunnyvale, CA). The fluorogenic substrate is specific for the chymotryptic activity of the proteasome and does not interfere with the tryptic or caspase-like activities of the organelle [22]. All measurements were performed in duplicate and were repeated in four independent experiments.

### Optical mapping

The membrane potential (V_m_) of the right atria was measured following optical mapping. The hearts from six-month old mice were perfused in the Langendorff mode and stained with 8 μl of V_m_-sensitive dye di-4-ANEPPS (0.5 mg/ml DMSO) by injecting the dye through a port on the bubble trap above the perfusion cannula. The fluorescence of di-4-ANEPPS was excited at 530 nm and emission collected at > 610 nm. The ventricular region in conjunction to the atrium was covered by a piece of blackout fabric (Thorlabs) to eliminate the interference from the ventricular V_m_. Blebbistatin (10 μM), an excitation-contraction uncoupler was applied to prevent motion artifacts. The optical V_m_ signals were recorded with a synchronized charge coupled device (CCD) camera (RM-6740CL, JAI) operating at 700 frames per second with a spatial resolution of 112 × 80 pixels using customer-developed software.

### Statistical Analysis

All data reported as mean± SEM of at least four independent experiments. Statistical analysis was performed with two-tailed ANOVA or Student’s *t* test using GraphPad Prism v6.01. Significance was assigned at P<0.05.

## Results

### SLNT5A replaces endogenous SLN in atria of TG mice

To determine the role of T5 in modulating SLN function in vivo, we transgenically overexpressed NF-SLNT5A in mice hearts using α-MHC promoter. We obtained two independent TG lines out of 28 initial F0 mice screened. These two TG lines were fertile and produced progenies. Pups from the TG mice breeding were born in the expected Mendelian ratio and were indistinguishable from their NTG control littermates.

**Fig 1:**
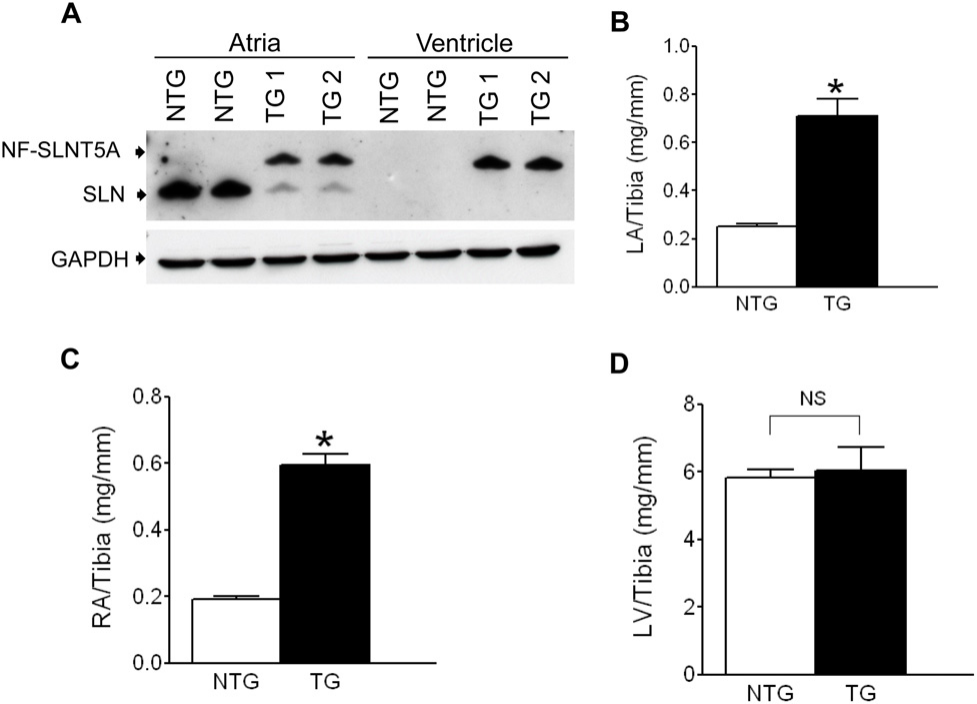
SLNT5A TG mice develop bi-atrial enlargement. **(A)** A representative Western blot showing similar levels of NF-SLNT5A protein in two-independent transgenic lines (n = 3). Morphometric analyses show that the ratios of LA to tibia length **(B)** and RA to tibia length **(C)** are significantly increased in the TG mice indicating bi-atrial enlargement. The ratio of LV weight to tibia length **(D)** is not significantly different between the NTG and TG mice. *Significantly different from the NTG mice. (p<0.0005), n = 6. NS-not significantly different.

To determine the expression levels of SLNT5A protein in the TG mice hearts, Western blot analysis was carried out. Results indicated that the SLNT5A protein levels in atria and in the ventricles of the two TG lines were indistinguishable (Fig. 1A). Since both TG lines have similar levels of transgene expression and showed similar phenotypes, we selected one of the TG lines for all other studies.

### Transgenic expression of SLNT5A is associated with cardiac pathology

We next examined the effect of SLNT5A expression on the cardiac morphology and structure. Morphometric analyses depicted that the left atrial (LA) weight to tibia length ratio (NTG- 0.25±0.01 vs. TG-0.71±0.07; g/cm; N = 8, p<0.0005) and the right atrial (RA) weight to tibia length ratio (NTG- 0.19±0.01 vs. TG-0.60±0.03; g/cm; N = 8, p<0.0005) were significantly increased in the TG mice (Fig. 1B & 1C) indicating a bi-atrial enlargement. The LV weight to tibia length ratio, however, was not significantly different between the NTG and TG mice (Fig. 1D).

**Fig 2:**
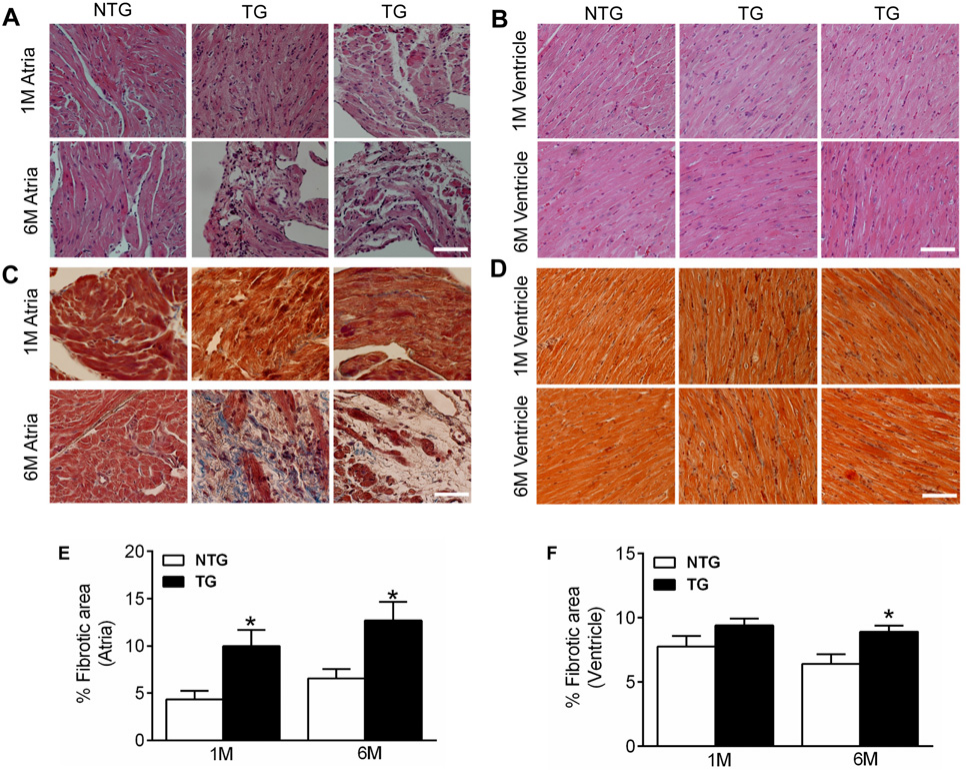
Cardiac structural remodeling in the SLNT5A TG mice. Representative sections from atria and the ventricles of one-month (1M) and six-month (6M) old TG mice stained with H&E (**A** and **B**) and trichrome (**C** and **D**). Fibrotic scar formation, collagen accumulation, myolysis and muscle disarray are progressive and are more prominent in 6M old TG mice hearts. Bar represents 50μm. Quantitation of fibrotic area in atria and in the ventricles are shown in panel **E** and **F** respectively.

To determine the structural remodeling, H&E (Fig. 2A &2B) and Masson’s trichrome (Fig. 2C & 2D) staining were carried out on one- and six- month old TG mice hearts. Results showed severe structural abnormalities such as fibrotic scar formation, collagen accumulation, myolysis and muscle disarray in atria and to a lesser extent in the ventricles of one- and six- month old TG mice. The quantitation of fibrotic area indicates that TG atria (Fig. 2E) underwent a more severe fibrosis than the ventricles (Fig. 2F). Further these changes were more prominent in six-month old TG mice hearts. There were no gender differences in cardiac morphometry and structural remodeling in the TG mice.

### Decreased expression of SR Ca^2+^ handling proteins in the TG mice hearts

**Fig 3:**
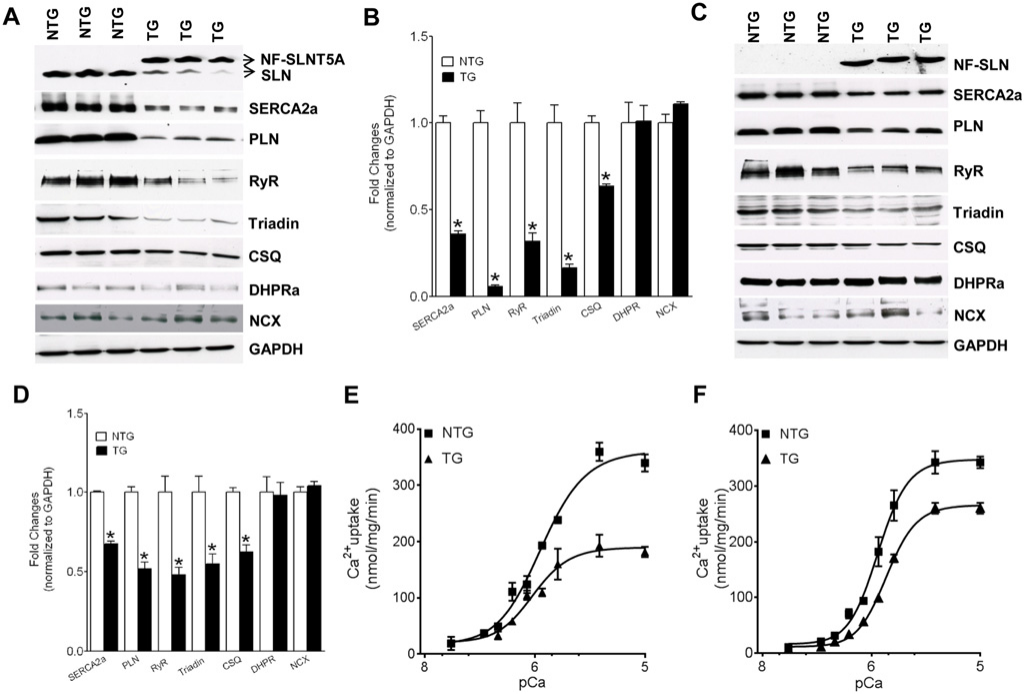
Selective downregulation of SR Ca^2+^ handling proteins and decreased SR Ca^2+^ uptake in TG mice hearts. Equal amounts of total protein prepared from the atrial **(A)** and ventricular **(C)** tissues of one-month old TG and NTG mice were separated on SDS-PAGE and immunoprobed with specific antibodies. Quantitation of signals by densitometry and normalization to GADPH levels shows selective downregulation of SR Ca^2+^ handling proteins in the TG mice atria **(B)** and ventricles **(D)**. *indicates the significant difference (p<0.05) between NTG and TG groups. n = 5. Calcium dependent SR Ca^2+^ uptake is significantly decreased in atria **(E)** and in the ventricles **(F)** of one-month old TG mice. For each atrial Ca^2+^ uptake experiment, atria from four mice were pooled. n = 4 for each group. The V_max_ of Ca^2+^ uptake was obtained at pCa 5.5.

Next, we determined the expression levels of Ca^2+^ handling proteins in the TG hearts by quantitative Western blot analysis. Since atrial remodeling is severe in six-month old TG mice, cardiac tissues from one-month old mice were used for this study. Results show that in the TG atria, the mutant SLN replaces the endogenous SLN without altering the total SLN content (SLNT5A = 86.3±0.72 vs. WTSLN = 13.6±0.7, % n = 5). The expression levels of other major SR Ca^2+^ handling proteins such as SERCA2a, PLN, RyR, triadin, and CSQ were significantly decreased in atria (Fig. 3A & 3B) and in the ventricles (Fig. 3C & 3D) of TG mice. Additionally, these changes were more prominent in atria than in the ventricles of TG mice. On the other hand, the level of sarcolemmal Ca^2+^ handling proteins, such as L-type Ca^2+^ channel subunit, DHPRα and NCX were unchanged in atria and in the ventricles of TG mice compared to that of age- and sex- matched NTG controls.

### Decreased maximum velocity of SR Ca^2+^ uptake in the TG hearts

The rate of Ca^2+^ dependent SR Ca^2+^ uptake was measured in atrial and ventricular homogenates from one-month old TG mice. Results showed that the Ca^2+^ dependent Ca^2+^ uptake was significantly reduced both in atria and in the ventricles of TG mice (Fig. 3E & 3F). The maximum velocity (V_max_) of SR Ca^2+^ uptake was significantly decreased in atria (NTG-339 ± 15 vs. SLN TG- 182 ± 9 nmol of Ca^2+^/mg of protein/min; N = 4; p<0.005) and in the ventricles (NTG-343 ± 20 vs. SLN TG- 260 ± 10 nmol of Ca^2+^/mg of protein/min; N = 4; p<0.05) of TG mice. Again these changes were more significant in atria than in the ventricles. The EC_50_ values calculated for the Ca^2+^ uptake were not statistically different between the TG and NTG mice hearts.

### Alterations in action potential and propagation in the TG mice atrium (optical mapping)

**Fig 4:**
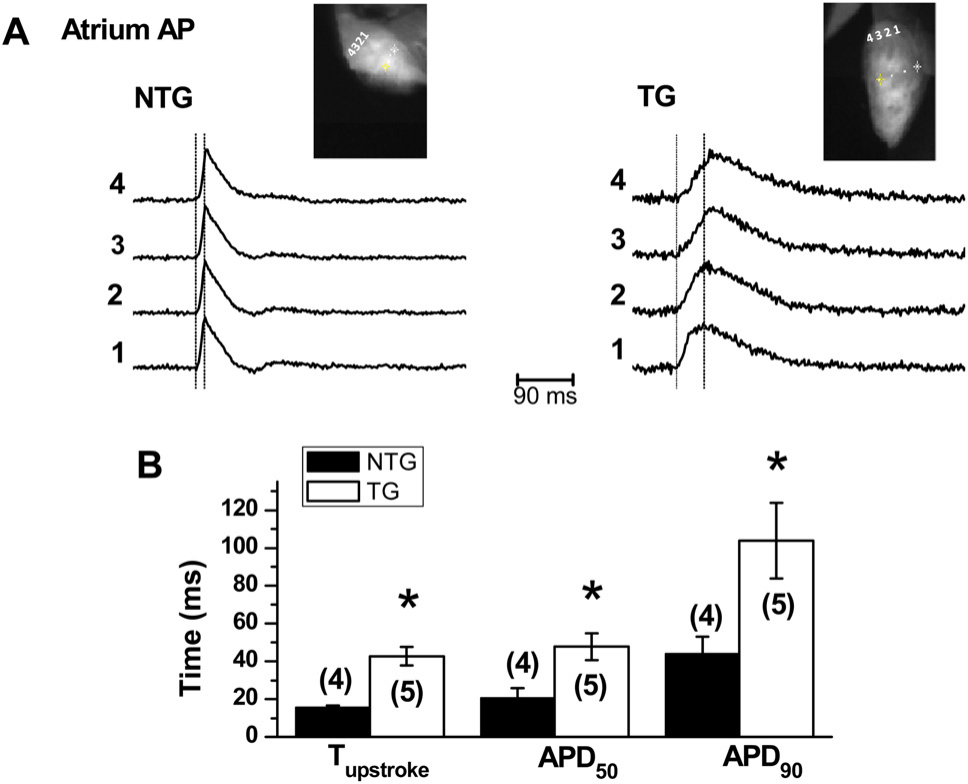
Optical APs recorded from right atria of six-month old TG and NTG mice hearts. **(A)** Representative traces recorded from the location points 1–4, respectively as indicated in the inset. The average upstroke period was indicated by the two vertical dashed lines in each panel. **(B)** Summarized values of upstroke time and AP duration at 50% (APD_50_) and 90% (APD_90_). *indicates the significant difference between TG and NTG groups (p<0.05).

To determine if the altered SR Ca^2+^ handling affected the electrophysiological function of atria, optical action potentials (APs) were recorded from the right atria of di-4-ANEPPS-loaded hearts of six-month old TG and NTG mice (Fig. 4). The duration of optical APs at 50% (APD_50_) and 90% (APD_90_) repolarization were longer in the TG mice atria (APD_50_ = 47.8 ±7.2 ms; APD_90_ = 103.9 ± 20.0 ms, n = 5) as compared to that of NTG controls (APD_50_ = 20.6 ± 5.2 ms; APD_90_ = 44.0 ± 9.1 ms, n = 4, p < 0.05). The depolarization time (upstroke) of optical AP was significantly longer in the TG mice atria (42.7 ± 4.9 ms, n = 5) relative to NTG controls (15.5 ± 1.2 ms, n = 4, p < 0.05), implicating a slower AP propagation in the TG mice atria.

### Decreased atrial contractility and diastolic dysfunction in the TG mice

Echocardiographic measurements on the two-month old mice show that there were no significant differences in the LV end-diastolic dimension (LVEDD), LV end-systolic dimension (LVESD), EF and FS between TG and NTG mice (Table 1). However, the EF and FS were higher in the six-month old TG mice compared to those of age- and sex- matched NTG control mice (Table 1). The diastolic septal wall thickness (DSEPWT) and systolic septal wall thickness (SSEPWT) were also increased in the TG mice, though these values were not statistically different from that of NTG control mice. Doppler echocardiography confirmed the enlargement of LA in six-month old TG mice. The atrial contraction velocity (A) however was significantly reduced in both two- and six- month old TG mice. The reduced “A” velocity resulted in a significant increase in the ratio of transmitral flow velocity during early diastolic (E) and atrial velocity, (E/A) in the TG mice (Table 1).

**Table 1:** Baseline echocardiographic data of TG and NTG mice.

Parameters	NTG, 2 month	TG, 2 month	NTG, 6 month	TG, 6 month
DSEPWT, mm	0.82±0.05	0.93±0.03	0.92±0.07	1.1±0.07
LVEDD, mm	3.76±0.15	3.76±0.05	3.54±0.1	3.52±0.24
DPWT, mm	0.69±0.02	0.79±0.04	0.95±0.1	1.15±0.08
SSEPWT, mm	1.11±0.03	1.3±0.08	1.27±0.11	1.58±0.07
LVESD, mm	2.41±0.11	2.48±0.05	2.48±0.09	2.19±0.16
SPWWT, mm	0.9±0.04	1.05±0.03*	1.26±0.11	1.48±0.11*
EF, %	74±1	71±1	66±2	76±1**
FS, %	36±1	34±1	30±1	38±1**
HR, bpm	417±17	359±17^#^	444±24	415±59
LA Diameter, mm	2.2 ± 0.05	2.8 ± 0.33	1.9 ± 0.1	3.5 ± 0.4**
E velocity, cm/s	68±2	69±8	58±8	68±2
A Velocity, cm/s	54±3	30±8**	44±6	18±3**
E/A ratio	1.3±0.1	2.8±1.0	1.4±0.1	4.4±0.6**

DSEPWT-diastolic septal wall thickness; LVEDD- left ventricular end diastolic dimension; DPWT- diastolic posterior wall thickness; SSEPWT- systolic septal wall thickness; LVESD-Left ventricular end systolic dimension; SPWWT-systolic posterior wall thickness; EF-ejection fraction; FS-fractional shortening; HR- heart rate; bpm- beats per minute; LA Diameter- left atrial diameter; E velocity- early filling velocity; A velocity- atrial filling velocity. Data are mean ± SEM.

**significantly different from NTG, p<0.005

*significantly different from NTG, p<0.05

n = 5 for 2 month old groups and n = 6 for 6 month old groups

^#^ NTG vs.TG

p = 0.056

**Fig 5:**
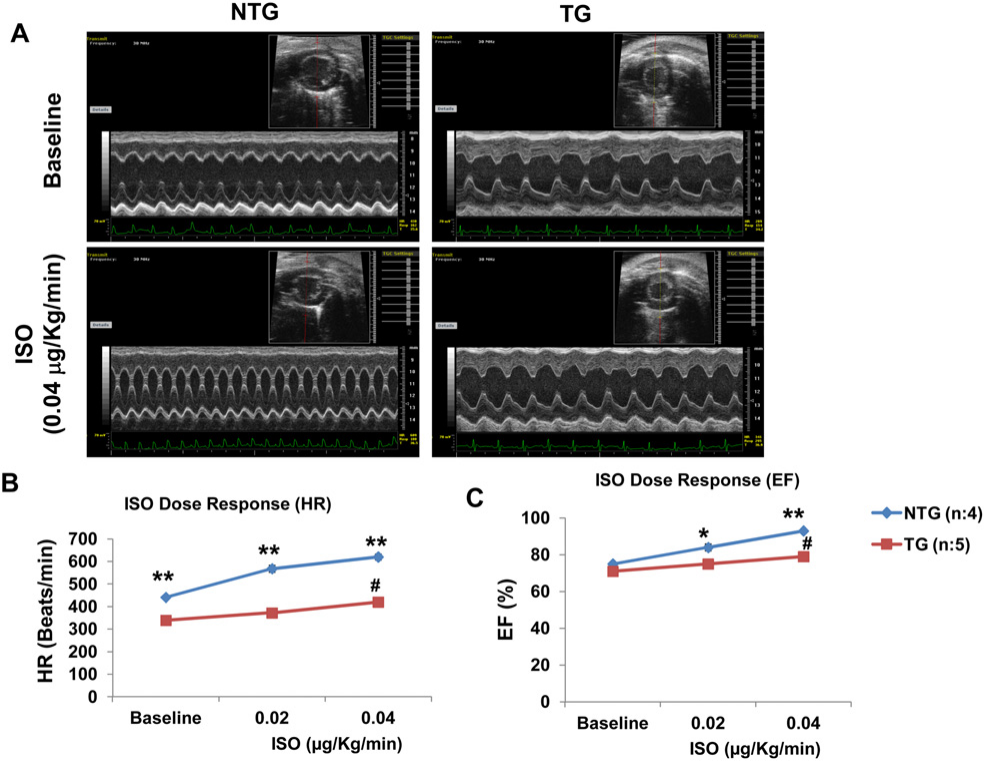
TG mice hearts show decreased ISO response. **(A)** Representative M-mode echocardiographic images of the left ventricle in NTG and TG mice at baseline and post ISO (0.04μg/Kg/min) infusion. **(B)** Dose response curves showing the changes in heart rate (HR) and **(C)** ejection fraction (EF) after infusion of ISO at 0.02 and 0.04 μg/Kg/min in the TG and NTG mice. *NTG vs. TG; p<0.05, ** NTG vs. TG; p<0.005, ^#^baseline vs. ISO (0.04 μg); p<0.05.

We next examined how the transgenic expression of phosphorylation defective mutant SLN affects the hearts ability to respond to β-adrenergic receptor stimulation. Results in Fig. 5 show that the NTG hearts responded to increasing doses of ISO (0.02 and 0.04 μg/Kg/min) with significant increase in frequency and contractility as shown by increased heart rate (HR) and EF. On the contrary, the TG hearts showed a significantly decreased ISO response in comparison to that of NTG controls. Furthermore in the TG mice, the basal HR [basal-339±15 vs. ISO (0.04μg) -420±12; beats/min] and EF [basal-71±1% vs. ISO (0.04μg) -79±3%] were significantly increased only after the highest dose of ISO infusion (0.04 μg/Kg/min).

Hemodynamic measurements (Table 2) on the TG mice show an increased LV end diastolic pressure (LVEDP; NTG = 3±1 vs. TG-11±1 mmHg, n = 4; p<0.005) and a decreased LV-dP/dt (NTG = 6450±703 vs. TG = 4550±210 mmHg, n = 4; p<0.05). The LV +dP/dt also decreased, but statistically not different (P = 0.06).

**Table 2:** Hemodynamics measurements from 6 month old TG mice.

Parameters	NTG	TG
LVSP, mmHg	106±11	91±5
LV +dP/dt, mmHg/sec	8100±928	5925±170^**#**^
LV-dP/dt, mmHg/sec	6450±703	4550±210^*^
LVEDP, mmHg	3±1	11±1^**^
SBP, mmHg	98±8	88±6
DBP, mmHg	71±9	60±4
MBP, mmHg	80±8	69±5
HR, bpm	490±25	438±3

LVSP-LV systolic pressure; LVEDP-LV end diastolic pressure;

SBP- Systolic blood pressure; DBP-Diastolic blood pressure;

MBP-Mean blood pressure; HR- Heart rate; bpm- beats per minute.

^#^ p = 0.06

*p<0.05

**p<0.005

n = 4

### The expression and the activity of ubiquitin-proteasome components are increased in the TG mice hearts

**Fig 6:**
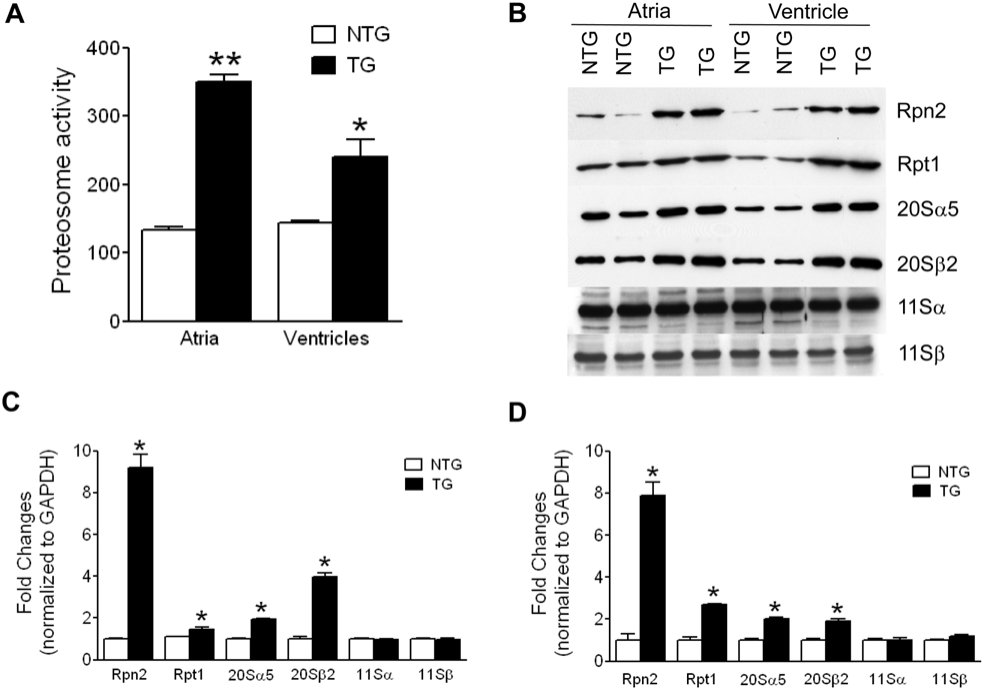
Activation of UPS in one-month old TG mice hearts. **(A)** Chymotryptic activity of the ubiquitin-proteasome in atria and in the ventricles of NTG and TG mice. *p<0.05; **p<0.005; n = 4. **(B)** Western blot analyses of 11S (11Sα and 11Sβ), 19S (Rpn2 and Rpt1) and 20S (20Sα5 and 20Sβ2) subunits of UPS. Bar diagram represents the quantitation of UPS components in atria **(C)** and in the ventricles **(D)** of TG and NTG mice. *indicates the significant difference between TG and NTG groups (p<0.05), n = 5.

Several studies have suggested that the ubiquitin-proteasome system (UPS) activation could contribute to the structural remodeling during cardiac pathology [21, 23–32]. We therefore determined if the cardiac structural remodeling is associated with the activation of UPS in the TG mice. Results in Fig. 6A show that the chymotrypsin-like activity of proteasomes was significantly increased in atria (p<0.001) and in the ventricles (p<0.05) of one-month old TG mice. To determine whether the increased ubiquitin-proteasome activity was due to the increased expression of UPS components, we measured the protein levels of 20S, 19S and 11S subunits. The quantitative Western blot analyses (Fig. 6B–D) indicated that the protein levels of both 20S (20Sα5, and 20Sβ2) and 19S (Rpt1, Rpn2) subunits were significantly (p<0.05) increased in atria and in the ventricles of the TG mice. The protein levels of 11Sα and 11Sβ subunits were unaltered in the TG hearts compared to that of NTG mice.

## Discussion

The major goal of this study is to determine the functional importance of threonine 5 in modulating SLN function in the heart *in vivo*. To achieve this goal, T5A mutant SLN was created to abrogate SLN phosphorylation and expressed in the mouse heart by transgenesis. Data from the studies on this TG mouse model demonstrated that the cardiac specific expression of SLNT5A results in: 1) a replacement of the endogenous SLN without changes in the total SLN content in atria, 2) cardiac structural remodeling and bi-atrial enlargement, 3) a reduction in the expression of SR Ca^2+^ handling proteins and Ca^2+^ uptake both in atria and in the ventricles, 4) a slower AP propagation and impaired atrial function, 5) a decreased ISO response and diastolic dysfunction, and 6) an activation of UPS in the heart.

Previously, we [3] and others [4] have demonstrated that overexpression of NF-wildtype SLN in mouse heart decreased the cardiac SERCA pump affinity for Ca^2+^ and the contractile function. Although one of these mouse models develops mild ventricular hypertrophy [4], both TG mouse models did not show any atrial pathology. On the other hand, the cardiac specific expression of SLNT5A results in severe atrial structural remodeling including collagen accumulation and cell necrosis, followed by atrial enlargement. These changes are progressive upon aging. Structural remodeling is also apparent in the TG ventricles but to a lesser extent in comparison with that of atria. In the TG atria, SLNT5A replaces the endogenous protein without altering the total SLN content. We also made similar observations in the atria of TG mice expressing NF-SLN (wildtype) [3], in which the NF-SLN replaces the endogenous SLN to maintain the total SLN content (data not shown). Thus, the atrial remodeling observed in the SLNT5A TG mouse model could be due to the functional consequences of mutant SLN rather than non-specific effect of transgene expression. At this juncture, it is also of interest to note that the cardiac specific overexpression of PLN impaired the atrial contractility but did not cause any atrial pathology [33, 34].

To determine the effect of SLNT5A on SERCA activity and Ca^2+^ transients at the cellular level, we attempted to isolate cardiac myocytes. However, due to the drastic cardiac structural remodeling, we were unable to isolate single cardiac myocytes feasible for Ca^2+^ imaging or patch-clamp studies. Biochemical studies have demonstrated decreased SR Ca^2+^ uptake in atria and in the ventricles of the TG mice. However, the selective downregulation of SERCA2a and other SR Ca^2+^ handling proteins in the TG hearts suggests that these changes could contribute to the decreased SR Ca^2+^ uptake. Because of these study limitations, we were unable to demonstrate the direct effect of SLNT5A on SERCA pump activity and/or Ca^2+^ transients. However based on data from the studies using isolated myocytes and heterologous cell systems [16, 17] and the impaired response of the SLNT5A TG hearts to β-adrenergic receptor stimulation (Fig. 5), we conclude that the T5A mutant SLN constitutively inhibits the cardiac SERCA pump and decreases the SR function. Further, these results suggest that SLN can be phosphorylated at T5 and is sufficient for mediating the cardiac responses to β-adrenergic stimulation.

Impaired SR function was shown to be compensated by changes in the sarcolemmal Ca^2+^ extrusion mechanisms [35] and these changes could contribute to the AP morphology and duration. In the TG hearts, we did not find changes in the expression levels of NCX or L-type Ca^2+^ channel proteins. However, the function of these channels as well as the expression and/or function of potassium channels may be altered and account for the overall prolongation of AP duration observed in the TG atria. The AP upstroke of depolarization, a major determinant of AP propagation in cardiac tissue was significantly slower in the TG atria. The downregulation of sodium channel expression and/or activation, as well as depolarized resting membrane potential level may contribute for these changes. Our future studies will address these mechanisms. In addition, the optical AP represents the sum of APs from multiple cells within a region of tissue. These results implicate a slower AP propagation in the TG mice atria.

Doppler echocardiographic data show reduced atrial filling (“A”) velocity in the TG mice. However, there is no significant difference in the early filling (“E”) velocity between NTG and TG mice. Thus the decreased “A” velocity along with the increased LVEDP could possibly cause a high E/A ratio observed in the TG mice. The basal systolic function of the heart is preserved in the TG mice. However, these hearts show decreased response to the β-adrenergic receptor stimulation. The reduced-dP/dt with increased LVEDP (Table 2) indicates impaired diastolic function in the TG mice. The increase in EF and diastolic dysfunction in the SLNT5A TG mice is unlikely to represent the most common forms seen in humans and thus it may simply reflect the consequence of mild ventricular hypertrophy as indicated by the increased diastolic and systolic post wall-thickness (Table 1) and fibrotic remodeling of TG ventricles.

Cardiac structural remodeling is a complex process and could be initiated by several signaling mechanisms. In pressure-overloaded cardiac hypertrophy and in myocardial ischemia, the UPS activation is shown to contribute to the ventricular remodeling [21, 23–32]. In our studies, we found that the activity and the expression levels of UPS components are increased both in atria and in the ventricle of one-month old TG mice. These changes are most prominent in atria than the ventricles and correlate with the extent of structural remodeling. The molecular mechanism which activates the UPS in the SLNT5A TG heart is not clear. It has shown that the UPS is activated during unfolded protein response due to ER stress[36]. Because ER function largely depends on Ca^2+^ homeostasis, it is tempting to speculate that the Ca^2+^ depletion in ER/SR of the TG mice hearts can induce the elevated expression of 19S and 20S components of the proteasome and its activity. The UPS activation might be a key secondary mechanism and have direct relevance for cardiac structural remodeling and subsequent atrial dilatation in the TG mice. Further, UPS activation may also specifically target and account for the decreased SR Ca^2+^ handling proteins. However, further studies are needed to validate these hypotheses.

In summary, our studies suggest that threonine 5 is the key amino acid that modulates SLN function in the heart in vivo. Furthermore, our studies suggest that alteration in SLN function can cause abnormal Ca^2+^ handling and subsequent cardiac remodeling and dysfunction.
